# Personalized, Naturalistic Virtual Reality Scenarios Coupled With Web-Based Progressive Muscle Relaxation Training for the General Population: Protocol for a Proof-of-Principle Randomized Controlled Trial

**DOI:** 10.2196/44183

**Published:** 2023-04-17

**Authors:** Susanna Pardini, Silvia Gabrielli, Silvia Olivetto, Francesca Fusina, Marco Dianti, Stefano Forti, Cristina Lancini, Caterina Novara

**Affiliations:** 1 Department of General Psychology University of Padova Padova Italy; 2 Digital Health Lab, Centre for Health and Wellbeing Fondazione Bruno Kessler Trento Italy; 3 Human Inspired Technology Research Centre University of Padova Padova Italy; 4 Padova Neuroscience Center University of Padova Padova Italy

**Keywords:** digital health, progressive muscular relaxation technique, mental well-being, virtual reality therapy, anxiety, relaxation, e-therapy, e-Health, virtual reality, tool, symptoms, depression, quality of life, coping

## Abstract

**Background:**

Virtual reality (VR) is an innovative tool that can facilitate exposure to either stressful or relaxing stimuli and enables individuals who have difficulties visualizing scenes to be involved in a more realistic sensorimotor experience. It also facilitates multisensory stimulation, a sense of presence, and achievement of relaxation. VR scenarios representing visual and auditory elements of natural relaxing environments can facilitate the learning of relaxation techniques such as the progressive muscle relaxation technique (PMRT). A complementary standardized technique deployed to reduce anxiety symptoms is the integration of PMRT and guided imagery (GI). Exposure to a pleasant imaginary environment helps the establishment of an association between a relaxing scenario and the relaxation technique, consequently promoting relaxation. Empirical evidence has shown that VR scenarios can increase the effects of relaxation techniques by enabling people to experience emotional conditions in more vivid settings.

**Objective:**

The main aim of this pilot study protocol is to investigate the impact on state anxiety of PMRT, associated with a personalized relaxing scenario in VR, and the role of VR scenarios in facilitating the recall of relaxing images and a sense of presence. A secondary aim is to understand if relaxing sessions administered via Zoom are more effective for managing anxiety and stress than a procedural setting based on audio-track guidance.

**Methods:**

Based on a longitudinal, between-subject design, 108 university students will be randomly exposed to one of three experimental conditions: (1) PMRT via Zoom and GI exposure, (2) PMRT via Zoom and personalized VR exposure, and (3) PMRT based on audio-track guidance and personalized VR exposure. Individuals are assessed before and after 7 training sessions based on self-report questionnaires investigating anxiety, depression, quality of life, coping strategies, sense of presence, engagement, and side effects related to VR exposure. Heart rate data are also detected by an Mi Band 2 sensor.

**Results:**

The experimental procedure is ongoing. In this paper, preliminary data from a sample of 40 participants will be illustrated. The experimental phase is expected to conclude in May 2023, and the final results of the research will be presented in June 2023.

**Conclusions:**

The results of this study will help shape the experimental design to apply it on a subsequent randomized controlled trial, also considering clinical samples. This work is expected to measure whether VR is a more engaging and helpful technique in promoting relaxation and decreasing anxiety levels than GI, by making the visualization process easier and by helping people to face more realistic sensory experiences. Assessing the efficacy of the PMRT in alternative delivery modes may extend its applications, especially in situations where the standard procedure is more challenging to be administered. To our knowledge, no equivalent study has been published so far on this matter.

**Trial Registration:**

ClinicalTrials.gov NCT05478941; https://clinicaltrials.gov/ct2/show/NCT05478941

**International Registered Report Identifier (IRRID):**

DERR1-10.2196/44183

## Introduction

### Background

Virtual reality (VR) is widely used to treat various mental diseases [1], also in association with different types of relaxation training (eg, progressive muscle relaxation training), and it facilitates multisensory stimulation, sense of presence, and achievement of relaxation [2,3]. VR can facilitate the learning of relaxation techniques through exposure to scenarios in which natural environments, which are commonly considered relaxing, are shown [2]. VR also has several advantages such as follows: easy to use, user-friendly, promotes engagement, and allows a full multisensory immersion in the virtual experience facilitating the control of external disturbing stimuli [4,5].

It was also highlighted that the customization of VR environments is an element that promotes relaxation. This latter type of user-centered approach [6] allows individuals to experience more realistic emotional conditions, like those experienced in everyday life, and it enhances relaxation, sense of presence, and perception of security in the virtual context (eg, [7]).

Standard relaxation programs usually include more directive techniques that imply larger involvement of the cognitive functioning system (eg, hypnosis) or the physiological response system (eg, progressive muscle relaxation technique [PMRT]), as well as fewer directive procedures that may be based on the cognitive functioning control (eg, meditation) or the physiological feedback (eg, autogenic training), respectively [8]. All these protocols are effective in promoting relaxation.

Jacobson [9] developed the PMRT protocol in 1938, suggesting that the overactivation of the sympathetic nervous system is primarily responsible for excessive skeletal muscle activity. Thus, interference in the skeletal muscle activity based on progressive relaxation reduces the activation. This process also positively affects other physiological parameters (eg, heart rate frequency) [10,11].

On these grounds, the training consists in learning how to tense and release several muscle groups.

Evidence suggests that stress-competing relaxation techniques are among the most effective training programs for enhancing psychological resources and reducing psychological distress [12].

PMRT is based on “top-down” and “bottom-up” neuronal processing. The “top-down” pathway is turned on by higher cognitive activities, such as focused attention, related to the cerebral cortex and the cerebellum, which send commands to peripheral areas by controlling muscle tension and gradual release. The holding and releasing activities allow proprioceptive stimulation from peripheral sensory afferents to the higher nervous system regions via the spinal cord and the brainstem as a “bottom-up” pathway [13-15]. Also, based on these processes, PMRT is considered one of the most effective and adaptable techniques for relaxation, anxiety management, stress, and depression reduction considering different kinds of target populations (eg, nonclinical individuals, patients with cancer, chronic pain, osteoarthritis, heart disease, chronic headache, anxiety, and depression) [16-20].

Still, the administration of combined methods appears very useful in reducing anxiety and the perception of pain in patients with medical conditions or during pregnancy [19-21]. Indeed, complementary methods that imply the administration of integrated cognitive and behavioral procedures are studied to manage anxiety, stress, depression, and pain, particularly in populations such as patients with cancer. Notably, the integration of PMRT and guided imagery (GI) enhances and facilitates the effect of relaxation, reducing anxiety and depression symptoms [19,22].

The application of GI, or VR, and PMRT is based on classical conditioning theory that involves learning relaxation techniques and exposure to relaxing mental images as conditioned stimuli useful for overcoming stress and specific fear [23,24].

The integration of PMRT and GI has been highlighted in the literature as an effective complementary method for reducing pain and anxiety symptoms (eg, [19,21]). GI is useful in creating mental imagery and refocusing attention on positive and relaxing imagined visual, auditory, tactile, or olfactory sensations, resulting in specific psychological and physiological responses, such as relaxation [25]. GI may also increase the reduction of the autonomic nervous system responses [19,25,26]. This combination can promote a higher sense of relaxation during training sessions, but it should also play a role in recalling a state of relaxation during everyday activities.

The combined methods introduced by Baird and Sands [19] implement eight steps [27-31]:

1. Setting a comfortable and protected real environment in which to relax
2. Recreating an imagined scene involving different senses
3. Guiding the user in focusing on breathing
4. Using passive progressive relaxation techniques
5. Recalling the imagined scene to direct the attention to pleasant stimuli
6. Feeling a release from pain and activation (eg, imagining a decrease in painful sensations)
7. Imagining moving into the scene by continuing to use focused breathing and relaxation
8. Ending the session (“Breathe deeply. When you are ready, allow the image to fade. Stretch and open your eyes” [19]).

Considering the positive impact on relaxation of GI associated with PMRT, exposure to a more vivid, closer-to-reality virtual experience should be a useful technique in further improving the relaxation learning promoted by the PMRT.

Furthermore, it is well known that before delivering PMRT with GI, some sessions of PMRTs, focused on learning to recognize and relax the muscle areas of our body, are needed. Usually, these training sessions are administered face-to-face in the therapist’s presence. Finally, to investigate the effectiveness of web-based psychotherapy interventions, there are controversial results regarding a PMRT procedure administered in vivo or via audio-tracks [32-34]. To our knowledge, no recent study exists addressing the comparison among training sessions administered in vivo, in a remote modality, or via audio-tracks. In light of the pandemic due to SARS-CoV-2, we intend to explore PMRT’s efficacy in a remote situation via both Zoom and audio-tracks.

In general, it is essential to point out that remote treatments have been increased. They promote users’ independence in managing some psychotherapy techniques, leaving time for other face-to-face psychotherapy activities that require the active involvement of both the patient and the therapist. Assessing the efficacy of PMRT in alternative settings may facilitate the administration of treatment where it is impossible to implement the standard procedure. It may also increase the positive impact of abbreviated relaxation session and combining relaxation methods with patients who have chronic pain or must be exposed to invasive medical treatments (eg, chemotherapy) [35,36].

### Study Overview and Objectives

The purposes of the present research project are as follows:

1. Investigating if PMRT associated with a personalized relaxing scenario in VR can facilitate relaxation and lower state anxiety
2. Understanding if the VR scenario helps recall the relaxing image assessed during the follow-up session
3. Investigating if VR is more effective in promoting the association between the relaxing scenario and PMRT than the standard procedure, consisting of PMRT associated with the imagery-induced exposure to a subjective relaxing environment
4. Analyzing if the relaxing sessions administered via Zoom are more effective in managing anxiety and stress than a procedure administered via audio-tracks.

We hypothesize that VR would be more effective than GI in promoting relaxation and decreasing state anxiety because it would facilitate the visualization process, allowing individuals to have more realistic sensory experiences than imagery-induced exposure.

## Methods

### Ethics Approval

This study protocol involving human participants was General Data Protection Regulation compliant and developed following the Declaration of Helsinki (Italian law 196/2003, European Union General Data Protection Regulation 679/2016). The institutional review board of the Interdepartmental Ethical Committee of Psychology (17 Area) of the University of Padova (Italy) approved the study protocol on May 28, 2021 (approval number 4213). In February 2022, the trial was tested based on a convenience sample of 5 individual volunteers from the general population. Then, it was decided to introduce the following questionnaires: the Coping Orientation to the Problems Experienced-New Italian Version (COPE-NVI), the ITC-Sense of Presence Inventory (ITC-SOPI), the Vividness of Visual Imagery Questionnaire [37,38], and the Test of Visual Imagery Control [37,38]. The amendments and supplementations have been accepted, and the protocol received the final approval of the institutional review board of the Interdepartmental Ethical Committee of Psychology (17 Area) of the University of Padova, Italy (approval number 4701; April 29, 2022). Before their study enrollment, the participants signed a written informed consent form based on a paper-and-pencil form, agreeing to participate in all the study sessions.

They were informed that (1) their data would be confidential, (2) they could omit any information they did not wish to give, and (3) they could withdraw from the study without providing any explanation.

### Eligibility, Recruitment of Study Participants, and Randomization

The recruitment phase started in May 2022. Study participants were recruited in Northeast Italy via social networking websites (eg, Facebook groups) and by providing information on the research during university lectures. Those interested in participating were asked to make an appointment with the investigators to participate in the first assessment phase (T0) in which the inclusion and exclusion criteria were evaluated, written informed consent was obtained, and a baseline evaluation was made.

Eligible participants were adults from the general population (18 years or older), native Italian speakers, owning a PC, and able to use a PC and smartphone.

Participants were excluded from the study if they had been diagnosed with a severe mental disorder or medical conditions that could hinder their participation in the study (eg, neuromuscular disorders, severe psychiatric or neurological disorders, and assumption of drugs that could interfere with heart rate assessment and the subjective relaxation state), or if a psychotherapeutic treatment was ongoing.

Eligible participants were randomly allocated to 1 of 3 experimental conditions based on a simple blinded randomization via an Excel (Microsoft Inc) file.

Two experimenters, trained in cognitive and behavioral psychotherapy, conducted the relaxation sessions, each administering to 50% of the sample of each group to control for possible biases related to the therapist’s personality and competence. The 2 therapists who administered the experimental procedure received the same PMRT training and were supervised by a senior researcher and psychotherapist supervisors.

### The Integrated PMRT and Virtual Environment Design

The virtual environment design and the hardware and software equipment are described in the paper by Pardini et al [39], in which the aim was to investigate the user experience, preferences, and engagement of the exposure to personalized, natural, and realistic VR scenarios.

Specifically, we adapted the procedure that Baird and Sands [19] introduced to propose an integrated intervention using different new technologies.

The protocol concerns the learning of the adapted abbreviated PMRT [33] in 5 sessions 2 times a week, as follows:

- Four sessions of active progressive muscle relaxation, in which participants are required perceptively learn the differences between the tension and relaxation of different muscle sections of the body. Each session has a duration of approximately 25 minutes. The first session is dedicated to the hands, forearms, arms, neck, shoulders, and back; the second to the facial muscles; the third to the diaphragmatic breathing; and the fourth to the abdomen, buttocks, and lower limbs. These sessions take place via the Zoom platform and are conducted by a cognitive-behavioral psychotherapist. Each Zoom session consisted of filling out 2 measures for the state anxiety and relaxation state based on the Moodle e-learning platform, sharing general standardized instructions for conducting relaxation and the rationale of the PMRT, guided relaxation aimed at relaxation of a dedicated body site, and filling out the same measures administered at the beginning of the session. A profile was created for each participant to access the Moodle platform. For the participants in condition “intervention B” (described in the following section) who participated in the first 4 PMRT sessions through the audio-track, all sessions were self-managed within the Moodle platform. Various activities have been scheduled based on the beginning of the training and the end of the activities planned in previous sessions.
- The fifth VR session is administered at the University of Padova Laboratory. All participants are asked to wear a smartwatch for heart rate detection. A total of 5 measurements (1 per minute) are made before the VR experience, 12 during the entire exposure in the virtual environment, and 5 after the experience in the virtual context. A duration of approximately 12 minutes has been established to avoid potential cybersickness symptoms during the virtual experience. This brief relaxation session is practiced by inexperienced individuals allowing for a decrease in anxiety and negative mood [40,41].

### Study Design

**Figure 1:**
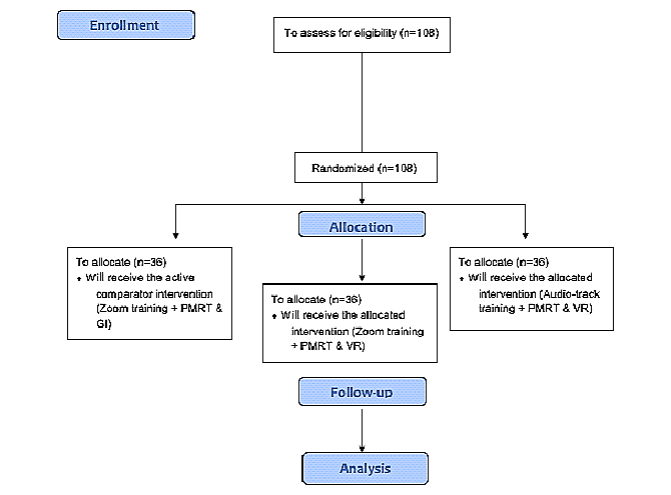
Consolidated Standards Of Reporting Trials 2010 flowchart. GI: guided imagery; PMRT: progressive muscle relaxation technique; VR: virtual reality.

**Figure 2:**
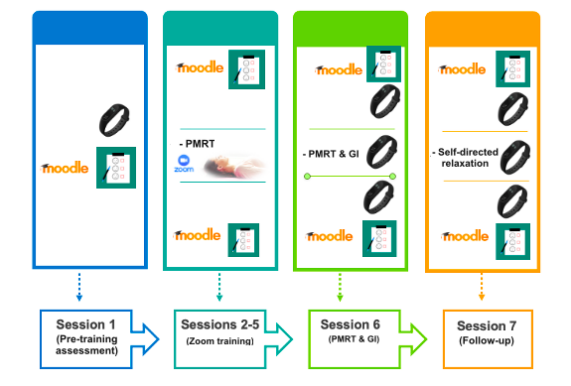
Graphic description of the activities carried out during the Active Comparator condition: progressive muscle relaxation technique (PMRT) training via Zoom and guided imagery (GI) exposure.

**Figure 3:**
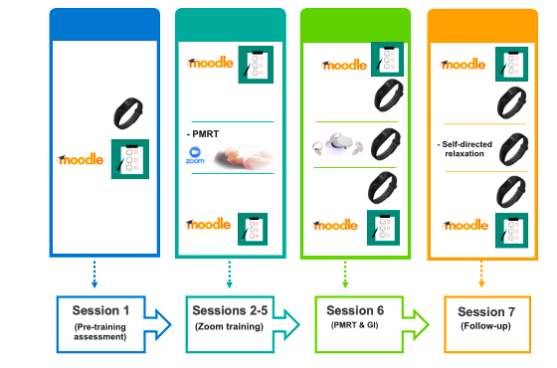
Graphic description of the activities carried out during the Intervention A: PMRT training administered via Zoom integrated with a personalized virtual reality exposure deployed by a head-mounted display (Oculus Quest 2). GI: guided imagery; PMRT: progressive muscle relaxation technique.

**Figure 4:**
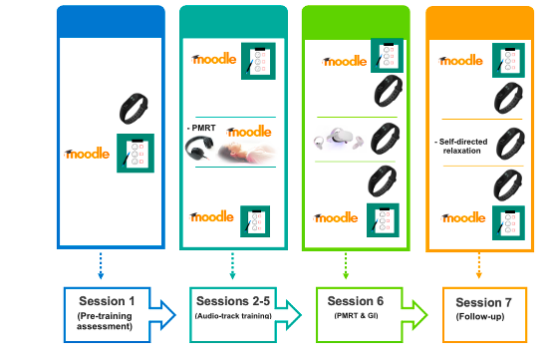
Graphic description of the activities carried out during the Intervention B: PMRT training administered based on the Moodle platform via an audio-track integrated with a personalized virtual reality exposure deployed by a head-mounted display (Oculus Quest 2). GI: guided imagery; PMRT: progressive muscle relaxation technique.

The present protocol of the proof-of-concept study is a longitudinal, between-subjects, 3-armed randomized controlled trial aiming to compare three treatment conditions (Figure 1):

1. Active Comparator condition, consisting of the deployment of the PMRT training via Zoom and GI exposure. It consists of a standard behavioral intervention based on 4 individual PMRT sessions via Zoom (t1-t4), an in vivo PMRT relaxing session and GI conducted by a psychotherapist (T1) after a week from the baseline assessment (T0), and a follow-up phase (T2) after 2 weeks consisting in recovering the GI relaxing scenario and PMRT session (see Multimedia Appendix 1 and Figure 2).
2. Intervention A is an experimental condition in which the complementary intervention is composed of the PMRT administered via Zoom integrated with a personalized VR exposure deployed by a head-mounted display (Oculus Quest 2). It comprises 4 individual PMRT sessions via Zoom (t1-t4), an in vivo PMRT relaxing session and VR exposure (T1) after a week from T0, and a follow-up phase that consists of the same activities as the Active Comparator condition (see Multimedia Appendix 1 and Figure 3).
3. Intervention B is an experimental condition in which the complementary intervention is composed of progressive muscle relaxation training administered through an audio-track integrated with the same personalized VR exposure setup in Intervention A. It is composed of 4 individual PMRT sessions via an audio-track delivered on the Moodle platform (t1-t4), an in vivo PMRT relaxing session and VR exposure (T1) after a week from T0, and a follow-up phase that consists of the same activities as the Active Comparator and the Intervention A conditions (see Multimedia Appendix 1 and Figure 4).

The assessment phases take place at baseline (T0), before and after the 4 PMRT sessions (via Zoom or audio-track), at the fifth in-presence session (T1), and a week later (T2).

The T0 (baseline) assessment phase has a duration of approximately 37 minutes, is the same for all participants, and is administered at the Virtual Reality Laboratory (A10-A11), Department of General Psychology, University of Padova (Italy). In this phase, the following measures are administered: (1) a demographic schedule (addressing age, gender, nationality, mother tongue, marital status, years of school attendance, employment status, psychological problems or disorders, ongoing psychological treatment, drug use, medical conditions, neuromuscular issues, previous injures, previous experiences in relaxation practice or anxiety management training, or with Oculus), and also, refraining from smoking, intense physical exercise, consuming caffeine for at least 1 hour before testing, and consuming alcohol for at least 6 hours before testing [42]; (2) a series of self-report questionnaires investigating depression, anxiety, stress, quality of life, and distress coping strategies (State Trait Anxiety Inventory-Y [STAI-Y], Depression Anxiety Stress Scales-21 [DASS-21], Psychological General Well-Being Index [PGWBI], and COPE-NVI); and (3) resting heart rate detection with an Mi Band 2 sensor.

Before and after each relaxation session (t1-t4), in approximately 20 minutes, the personal level of tension is assessed by using a visual analog scale from 0 (no tension) to 10 (extreme tension level). The state-anxiety level is evaluated based on the STAI-Y1. The 4 relaxation sessions are administered 2 to 3 days apart from each other for all 3 groups. The assessment phase is administered through Moodle, an e-learning platform used for data collection.

The T1 phase (day 7) required approximately 60 minutes. Before and after the relaxation session, states of tension and anxiety are assessed by using a 0 (no tension) to 10 (extreme tension level) scale, and the state-anxiety level is evaluated based on the STAI-Y1. Then, participants are exposed to a PMRT session merged with a VR or a GI procedure. Before the GI or the VR experience, all the participants fill out the Vividness of Visual Imagery Questionnaire and the Test of Visual Imagery Control. After the PMRT session, users compile a series of self-report questionnaires investigating depression, anxiety, stress, and quality of life (STAI-Y, DASS-21, PGWBI), and the VR group only fill out the Virtual Reality Sickness Questionnaire to monitor VR-related side effects (eg, sickness) and the ITC-SOPI to assess the sense of presence at the end of the T1 phase. The Mi Band 2 sensor is used during the entire T1 phase administration to detect resting heart rate activity. This assessment phase is administered through the Moodle e-learning platform.

The T2 phase (follow-up) is deployed for approximately 45 minutes. Before and after the relaxation session, states of tension and anxiety are assessed using a visual analog scale from 0 (no tension) to 10 (extreme tension level). The state-anxiety level is evaluated based on the STAI-Y1. All the users are exposed to a self-GI experience in which those who were part of the VR group are asked to recall the personalized VR scenario experienced during the T1 phase (day 7). Instead, the GI group retrieve the image participants had used in association with the PMRT during the T1 phase (day 7). After the session, participants fill out a series of self-report questionnaires investigating depression, anxiety, stress, quality of life (STAI-Y, DASS-21, PGWBI), and an ad hoc version based on the ITC-SOPI to assess the sense of presence experienced during the imagery experience. This assessment phase is administered based on the Moodle e-learning platform. The Mi Band 2 sensor is used during the entire T1 phase administration (day 7) to detect resting heart rate activity.

### Power Calculation

Sample size estimation was computed with G*Power 3.1 software (Heinrich Heine Universität Düsseldorf) [43]. As a statistical test, the repeated-measures ANOVAs between factors have been considered. The effect size was set as 0.25, the α as .05, and the Power (1β error probability) as 0.80. We have 3 separate groups and 3 measurements. On the basis of these parameters, we estimated having to recruit at least 36 participants for each of the 3 groups.

### Data Collection, Storage, and Security

Based on informed consent, participants are informed about the experimental procedure; how data are collected, transmitted, and stored; and who has access to the data. Any digital data, such as responses to web-based questionnaires, are uploaded and saved to a secure server. Any hard copy documents are stored in a locked cabinet. Both digital data and hard copy documents are retained for 10 years after the end of the study and then securely destroyed.

### Measures-Screening and Primary Outcomes

In Table 1, all the assessment and primary outcome measures are described.

**Table 1:** Evaluation tools and methods by outcome of interest.

Primary objective	Description	Evaluation tool
1. Number of participants with virtual reality (VR)–related side effects as assessed by the Virtual Reality Sickness Questionnaire (VRSQ) and the investigators’ observations^a^	Participants in the 2 VR experimental arms (Interventions A and B) are asked to refer to whether the VR head-mounted display was painful, too heavy, or uncomfortable. Participants are asked if they have experienced negative side effects during the VR exposure based on the VRSQ, administered at the end of the VR exposure. This questionnaire is based on a dichotomous answer (Yes/No). During the VR experience, the investigator observes participant reactions and records the behaviors and signs that suggest an unpleasant experience.	VRSQ [44,45]
2. Changes between and within subjects in depression scores^b^	Differences between groups and change from baseline to the T1 and T2 phases in depression scores are assessed based on the Depressive Anxiety Stress Scale-21 (DASS-21-Depression Subscale). Items are answered using a 4-point Likert scale (0-3). Scores range from 0 to 42 (the scores will need to be multiplied by 2 to calculate the final score). Higher scores indicate more severe depression.	DASS-21-Depression Subscale [46-48]
3. Change is being assessed between and within subjects in trait-anxiety scores^b^	Differences between groups and change from baseline to the T1 and T2 phases in trait-anxiety scores are assessed based on the State-Trait Anxiety Inventory-Y2 (STAI-Y2, Trait Anxiety). The measure is a self-report questionnaire investigating trait anxiety based on a 4-point Likert scale (1-4). Scores range from 20 to 80. Higher scores indicate higher trait-anxiety severity.	STAI-Y2 [49-51]
4. Changes between and within subjects in state-anxiety scores^b^	Differences between groups and change from baseline to the T1 and T2 phases in state-anxiety scores are assessed based on the State-Trait Anxiety Inventory-Y1 (STAI-Y1, State Anxiety) before and after each session. The STAI-Y1 is a self-report questionnaire investigating the state anxiety based on a 4-point Likert scale (1-4). Scores range from 20 to 80. Higher scores indicate greater state-anxiety severity.	STAI-Y1 [49-51]
5. Change is being assessed between and within subjects in autonomic arousal, skeletal muscle effects, situational anxiety, and subjective experience of anxious affect^b^	Differences between groups and change from baseline to the T1 and T2 phases in anxiety scores are assessed by the “DASS-21-Anxiety Subscale” based on a 4-point Likert scale (0-3). Scores range from 0 to 42 (the scores will need to be multiplied by 2 to calculate the final score). Higher scores indicate more anxiety severity.	DASS-21-Anxiety Subscale [46-48]
6. Change is being assessed between and within subjects in bodily anxiety sensations^b^	Differences between groups and change from baseline to the T1 and T2 phases in bodily anxiety sensations scores are assessed based on the Self-Assessment Manikin (SAM). The SAM is a nonverbal imagery-based assessment technique used to assess bodily anxiety sensations that directly measure the pleasure, arousal, and dominance associated with a person’s affective reaction to a wide variety of stimuli. Each sensation is assessed on 5 different images distributed in order of size. Participants can tick either of the 5 pictures or 4 spaces in between, making it a 9-point Likert scale. The SAM varies from a minimum score of 3 to a maximum score of 27.	SAM [52]
7. Change is being assessed between and within subjects in levels of chronic nonspecific arousal (stress)^b^	Differences between groups and change from baseline to the T1 and T2 phases in levels of chronic nonspecific arousal (stress) scores are assessed by the “DASS-21-Stress Subscale” based on a 4-point Likert scale (0-3). Scores range from 0 to 42 (the score will need to be multiplied by 2 to calculate the final score). Higher scores indicate a higher level of stress.	DASS-21-Stress Subscale [46-48]
8. Change is being assessed between and within subjects in relaxation levels^b^	Differences between groups and change from baseline to the T1 and T2 phases in relaxation levels are assessed based on the visual analog scale administered before and after each session. This measure assesses relaxation levels before and after each VR experience. Participants had to express how relaxed they felt (0 =not at all relaxed; 10=completely relaxed; 0 =absent; 10=complete). Higher scores indicate higher relaxation levels.	Visual analog scale-relaxation level
9. Change is being assessed between and within subjects in quality-of-life scores^c^	Change from baseline to the T1 and T2 phases in quality-of-life scores on the Psychological General Well-Being Index (PGWBI) is assessed at 3 different assessment times. The measure consists of 22 self-administered items, rated on a 6-point scale (0-5), which assesses psychological and general well-being. Scores range from 0 to 110. Higher scores indicate greater well-being.	PGWBI [53-55]
10. Change is being assessed between and within subjects in coping strategies scores^b^	Change from baseline to the T1 and T2 phases in coping strategies is assessed at 3 different assessment times. The Coping Orientation to the Problems Experienced-New Italian Version (COPE-NVI) is a self-report questionnaire developed to assess the different coping strategies people use in response to stress; it is based on 60 items rated on a 4-point scale (1-4). Total scores range from 60 to 240. Higher scores indicate that a certain coping strategy is frequently used by the person.	COPE-NVI [56]
11. Change is being assessed between and within subjects in sense of presence scores related to the VR and guided imagery experiences^c^	Differences between groups and change from the T1 and T2 phases in sense of presence related to the VR or guided imagery experience are assessed at 2 different assessment times based on a visual analog scale and the ITC-Sense of Presence Inventory (ITC-SOPI). The ITC-SOPI is a self-report questionnaire assessing the sense of presence. Items are rated on a 5-point Likert scale (1-5). Scores range from 36 to 180. To obtain the scores for each of the scales, the average of the items is calculated. Higher scores indicate a higher sense of presence.	ITC-SOPI [57]; Visual analog scale-sense of presence
12. Change is being assessed between and within subjects in heart rate^b^	Differences between groups and change from baseline to the T1 and T2 phases in heart rate are assessed based on the Xiaomi Mi Band 2 sensor. It is used in 3 different assessment times (T0, T1, T2). During T1 and T2 heart rate is measured before, during, and after the in-presence relaxation session.	Xiaomi Mi Band 2 tool [58,59]

^a^T1 (day 7) phase.

^b^T0 (baseline), T1 (day 7), and T2 (day 14) phases.

^c^T1 (day 7) and T2 (day 14) phases.

### Statistical Procedure and Data Analysis

Quantitative statistical analyses will be performed using SPSS (versions 27.0 and 28.0; IBM Corp) [60,61] and R package [62]. To investigate the normal data distributions of the dependent variables, ranges of skewness and kurtosis will be determined. The Kolmogorov-Smirnov and the Shapiro-Wilk tests will also be performed to evaluate the statistical significance (mean, 95% CI) and normality of the distributions. In addition, Cronbach α will be assessed for each self-report questionnaire’s subscales. To explore sociodemographic features, frequencies, means, and SDs will be measured.

To examine differences between and within subjects, the 3 groups will be evaluated by a repeated-measures ANOVA for mixed designs, where individuals will be the random effect, while the type of intervention and time (T0/T1/T2/before and after each session) will be the fixed effects. The Akaike information criterion and binary logistic regression will be performed to explore significant influence factors.

Spearman rank correlation coefficient will be used to investigate the relationship between the self-reported measures and heart rate.

## Results

As of July 2022, a total of 45 individuals have been assessed as eligible for study enrollment (Multimedia Appendix 2).

Preliminary data showed adequate Cronbach α for all the T0 self-report questionnaire scores (ranging from .66 to .83). No differences emerged between the GI and VR groups at the baseline (T0) for all the sociodemographics and psychological features investigated (gender, marital status, school attendance, employment, participation in relaxation training in the past, use of VR in the past, anxiety, depressive and stress symptomatology, and the ability to imagine in a vividness way mental images) except for age (*F*_1,39_=12.32; *P*<.001) and years of education (*F*_1,39_=4.81; *P*=.04), factors that have been controlled for in the following group comparisons. To preliminarily investigate the differences between the VR and GI groups in experiencing state anxiety before and after the relaxation session (T1) and at T2, repeated-measures multivariate ANOVAs have been computed. At T1, our preliminary analysis showed a significant difference of the state-anxiety symptoms (*F*_1,39_=22.48*;* *P*<.001) between groups with lower scores in state anxiety (VR group: mean 30.10, SD 4.35; GI group: mean 39.35, SD 4.71; *t*_1,39_=5.92*;* *P*<.001) for the VR group after the relaxation session. Moreover, the VR group perceived a higher sense of physical presence (*F*_1,39_=7.38*;* *P*<.01). The study’s experimental phase is expected to conclude in May 2023, and the results of the research will be presented in June 2023. The results obtained from this work will be published in an open-access scientific paper and in the form of posters and conference papers to be presented at national and international conferences.

## Discussion

This study aims to evaluate the impact of exposure to customized natural and realistic VR-based scenarios as a factor that may enhance relaxation when administered with PMRT. To our knowledge, few studies have addressed this topic [63].

Furthermore, in light of the COVID-19 pandemic, innovative VR treatment protocols are increasingly requested and helpful in managing relaxation, also due to the possibility to be administered with larger flexibility in several different contexts and partially independent from the presence of the therapist [64].

Moreover, delivering an entire PMRT protocol with a psychotherapist present may require a considerable amount of time and higher costs. The introduction of VR may positively reduce intervention costs, as it allows partial self-management of the treatment. Furthermore, in the case of patients with medical problems (eg, cancer), the integration of VR may facilitate the administration of the relaxation intervention during specific invasive treatments (eg, chemotherapy sessions).

Assessing the efficacy of PMRT in alternative ways could extend the treatment administration, especially in situations where the standard delivery procedure is more difficult to implement.

With the aim to preliminarily investigate this study’s main objective, initial analyses showed that VR with PMRT facilitates a greater decrease of state anxiety than GI. Based on our knowledge, no studies have investigated VR with PMRT as an alternative complementary technique instead of GI. The current findings are promising and will be extensively investigated at the end of the recruitment phase.

This study is characterized by a series of limitations that should be considered and solved in the implementation of future studies. First of all, it is essential to consider the prominent role that psychophysiological parameters have in measuring relaxation, defined as the ability of the human body to reduce physiological arousal, and which can be assessed by measuring a decrease in muscular tension, heart rate, respiration rate, and sweating rate [3,65]. Although our project is targeted at investigating a self-reported sense of presence, engagement, and relaxation feeling, it should be important to also investigate psychophysiological measures other than heart rate, such as breathing rate, heart rate variability, galvanic skin response, cortical levels, and changes in brain wave activities. Research conducted so far investigated these parameters to understand the impact of distinct VR-based relaxation techniques different from progressive muscle relaxation, showing promising results [3]. Moreover, it would be important to thoroughly investigate psychophysiological activity also when VR is merged with PMRT both with clinical and nonclinical samples.

## Appendix

Multimedia Appendix 1 SPIRIT (Standard Protocol Items: Recommendations for Interventional Trials) diagram. Overview of the enrollment, interventions, and assessment phases.

Multimedia Appendix 2 CONSORT 2010 flowchart.

## Abbreviations

COPE-NVI: Coping Orientation to the Problems Experienced-Nuova Versione Italiana
DASS-21: Depression Anxiety Stress Scales-21
GI: guided imagery
ITC-SOPI: ITC-Sense of Presence Inventory
PGWBI: Psychological General Well-Being Index
PMRT: progressive muscle relaxation technique
STAI-Y: State-Trait Anxiety Inventory-Y
T0: baseline
T1: the fifth in-presence session
T2: the last session
VR: virtual reality

## Data Availability

The data set generated and analyzed during the current study is available from the corresponding author on reasonable request.
