# Supplementary figures and images for: Personalized, Naturalistic Virtual Reality Scenarios Coupled With Web-Based Progressive Muscle Relaxation Training for the General Population: Protocol for a Proof-of-Principle Randomized Controlled Trial
Source: JMIR Res Protoc. 2023 Apr 17;12:e44183. doi: 10.2196/44183 (PMC10152380; doi:10.2196/44183)

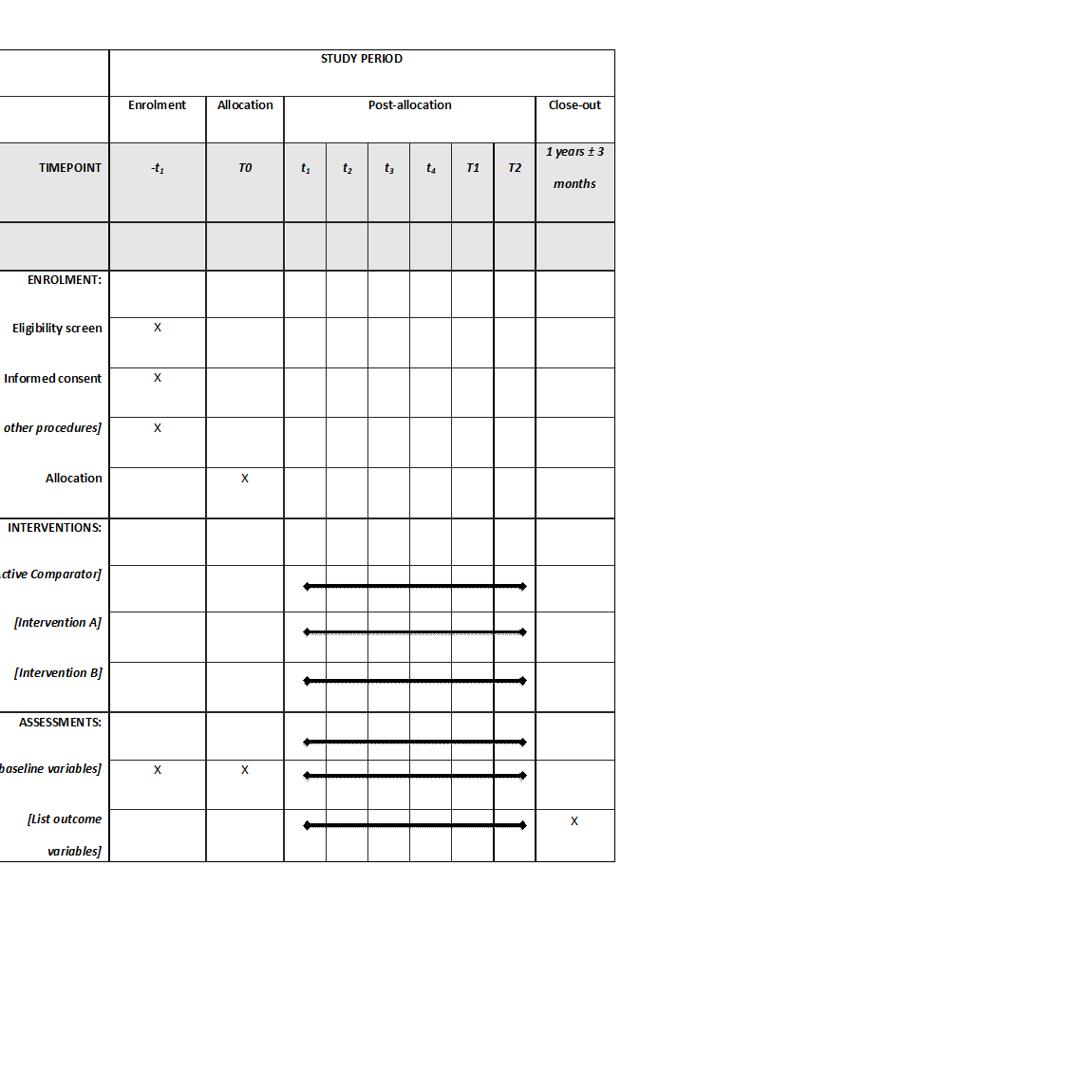

Supplement: Multimedia Appendix 1 [file resprot_v12i1e44183_app1.png]

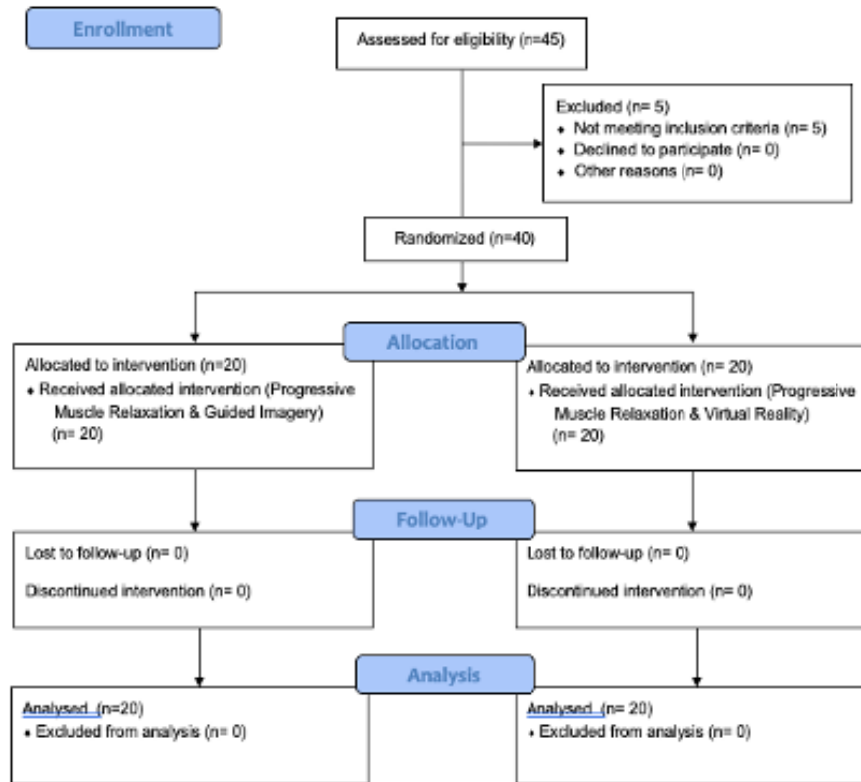

Supplement: Multimedia Appendix 2 [file resprot_v12i1e44183_app2.pdf]
